# Getting under the skin: resident memory CD8^+^ T cells have a second residence in the draining lymph node

**DOI:** 10.1038/s41435-024-00266-7

**Published:** 2024-03-08

**Authors:** Teresa Neuwirth, Azuah L. Gonzalez, Emilie Fisher-Gupta, Georg Stary

**Affiliations:** 1 Center for Molecular Medicine of the Austrian Academy of Sciences, Vienna, Austria; 2 Department of Dermatology, Medical University of Vienna, Vienna, Austria; 3 Department of Molecular Pathology and Immunology, Vanderbilt University School of Medicine, Nashville, TN USA

**Keywords:** Lymphocyte activation, Lymphocyte differentiation, Immunological memory

Memory T cells, which form after primary infection, play an important role in achieving long-term adaptive immune protection and immunosurveillance against recurring infections. Different memory CD8^+^ T cell populations are thought to contribute to cellular immunity at different anatomical locations: central memory (T_CM_) and effector memory T cells (T_EM_) patrol lymphoid organs and blood to provide global surveillance of pathogens, while resident memory T cells (T_RM_) predominantly reside in non-lymphoid tissues (NLT), which are common sites of pathogen re-encounter [1]. In both mice and humans, T_RM_ are characterized by a high expression of the surface markers CD103 and CD69, which is known to downregulate the lymph-homing receptor S1PR1 and low expression of CD62L [2–5]. Phenotypically, T_RM_ are thought to be similar to recently activated effector T cells as they have increased cytotoxic potential and activate local immunity through cytokine production in contrast to T_CM_ which are re-activated in the secondary lymphoid organs [1]. This has led many to believe that T_RM_ represent a terminally differentiated population of memory T cells permanently anchored in the tissue with little to no potential for recirculation. Over the last decade, it has become increasingly clear that T cells with a T_RM_-like phenotype are not entirely restricted to NTLs, but can also be found in secondary lymph organs [6–8].

Classically, primary and memory T cell responses are thought to be initiated in the lymph nodes from where T cells migrate into peripheral tissue sites. This model is referred to as an ‘inside-out’ model and has more recently been challenged by multiple studies [7, 9, 10]. While this model holds true for the initiation of a primary immune response, it does not align with the behavior seen in T_RM_. In contrast to other memory T cell populations, T_RM_ have high proliferative potential leading to the local expansion of memory cells upon antigen restimulation [9]. Moreover, T_RM_ may rather follow an ‘outside-in’ model of retrograde migration. This suggest that T_RM_ can initiate a secondary immune response within tissues and then migrate to the draining lymph nodes (dLN). The presence of LN T cells with a T_RM_ phenotype was first observed upon LCMV infection, by which parabiosis experiments indicated their tissue residency [6]. LN T_RM_ show similar transcriptional profiles to NLT T_RM_ and share common surface marker expression with NLT T_RM_. The formation of LN T_RM_ depends on NLT T_RM_, which function as precursors to LN T_RM_, further supporting an ‘outside-in’ model of adaptive memory. However, how and when this migration from the tissue to the LN is initiated and how they re-migrate during a secondary infection has been unclear so far.

**Fig. 1:**
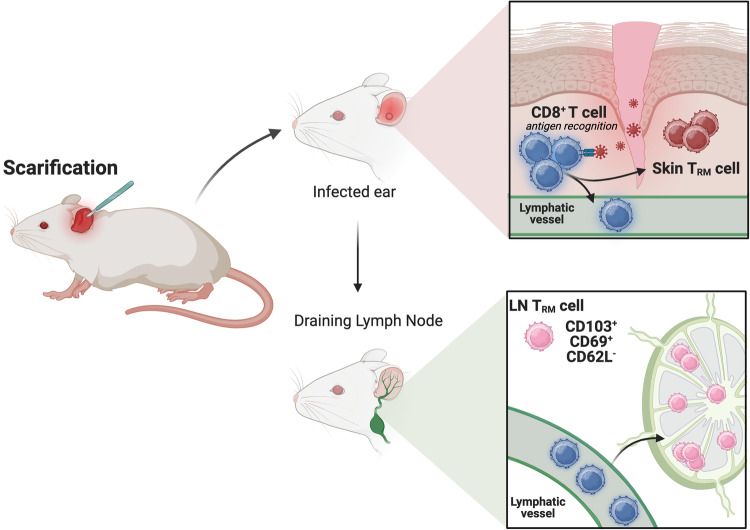
CD8^+^ T cells seed skin and lymph node T_RM_ after infection via the dermal lymphatic vessels. After infection with VV, CD8^+^ T cells in the skin recoginze viral antigens and subsequently seed skin T_RM_ and LN T_RM_. During the effector phase, lymphatic-dependent egress of LN T_RM_ is essnetial for LN T_RM_ seeding. Moreover, LN T_RM_ has a protective role upon rechallenge. Created with BioRender.

The preprint by Heim et al. [11] utilized a murine scarification model with vaccinia virus (VV) for a highly localized, self-resolving skin infection to study the ontogeny and developmental cues needed to seed resident memory CD8^+^ T cell populations in the skin and the dLN (Fig. 1). In line with previous reports, LN T_RM_ form exclusively in the dLN upon VV infection and exhibit high phenotypic similarity to skin-resident counterparts such as the expression of traditional skin T_RM_ markers CD69, CD103, and CXCR6. Moreover, skin T_RM_ and LN T_RM_ develop concurrently and remain randomly-distributed in the dLN up to 100 days after infection. Since skin-resident T_RM_ may be precursors for LN T_RM_, the authors utilized a mouse model expressing a soluble K14-driven VEGFR3-Ig fusion protein, which leads to the loss of dermal lymphatic vessels by trapping free VEGF-C, to address whether the lymphatics are required for seeding LN T_RM_. While there were no changes in skin T_RM_ abundance after VV infection, there was almost a complete ablation of LN T_RM_. These data suggest that dermal lymphatics are essential for the formation and seeding of LN T_RM_ in the dLN. Using Kaede transgenic mice, which express a photoconvertible fluorescent protein, the authors tracked skin-emigrating T_RM_ and found that LN T_RM_ seeding primarily occurs within the first 5 days after VV infection of the ear, further supporting the notion that egressing, effector CD8^+^ T cells represent a potential precursor population that seeds LN T_RM_.

To determine whether transit through the skin is necessary for LN T_RM_ development, the authors first depleted circulating T cells by injecting their mice with an anti-CD90.1 antibody, leaving skin T_RM_ intact. This was then followed by surgically removing the infected ear at different timepoints to physically prevent migration from the skin to the LN. In this setup, few LN T_RM_ were formed if the ear was resected at day 5 or later compared to non-resected controls. These data suggest that LN T_RM_ exclusively migrate from skin to the dLN. This was further corroborated in subsequent experiments where both ears from the same mouse were infected with VV and only the non-resected infected ear was able to form LN T_RM_. Together, this supports the notion that T cell egress from the skin is necessary for LN T_RM_ formation. In addition, adoptive transfer of TCR-reporter T cells (Nur77-GFP) revealed that TCR triggering occurs almost exclusively in the skin and not in the dLN, even after re-challenge. Lastly, the authors demonstrated that LN T_RM_ are primed for cytotoxicity and support the control subsequent infections. Collectively, this study highlights that T_RM_ transit from the skin to the dLN in the effector phase and derive from effector T cells rather than established skin T_RM_.

Overall, this preprint fills a gap in the mechanism of retrograde migration after skin infection by showing that dermal lymphatic vessels are required for LN T_RM_ seeding. However, the exact environmental clues mediating which T_RM_ leaves the skin to seed the LN are still unknown and warrant further investigation. Additionally, it shows how antigen encounter does not happen in the dLN for these T_RM_ but rather in the skin, providing additional evidence for an ‘outside-in’ model of T_RM_ -mediated immune memory. This study adds to the dogma that not only effector and central memory can be assessed by looking at secondary lymphoid tissues but that these organs do in fact, at least partly, represent the peripheral immune memory in NLT.
